# Confirmation of Di(2-ethylhexyl) phthalate-induced micronuclei by repeated dose liver micronucleus assay: focus on evaluation of liver micronucleus assay in young rats

**DOI:** 10.1186/s41021-024-00311-x

**Published:** 2024-08-23

**Authors:** Miyuki Shigano, Rie Takashima, Kensuke Satomoto, Henri Sales, Ryoko Harada, Shuichi Hamada

**Affiliations:** 1 Safety Assessment Department, Kashima Laboratories, Mediford Corporation, 14-1 Sunayama, Kamisu-shi, Ibaraki, 314-0255 Japan; 2 Nippon Kayaku Co., Ltd., 2-1-1 Marunouchi, Chiyoda-Ku, Tokyo, 100-0005 Japan; 3 Ishihara Sangyo Kaisha Ltd., 2-3-1 Nishi-Shibukawa Kusatsu-Shi, Shiga, 525-0025 Japan; 4 ITR Laboratories Canada Inc., 19601 Clark Graham Ave, Baie-D’Urfé, Quebec, H9X 3T1 Canada; 5 BoZo Research Center Inc., 1-3-11 Hanegi, Setagaya-ku, Tokyo, 156-0042 Japan

**Keywords:** Micronucleus, Liver, Phthalate, In vivo, Carcinogen, Rats

## Abstract

**Background:**

Di(2-ethylhexyl) phthalate (DEHP) is a plasticizer commonly used in a wide variety of products, including medical devices. It is rapidly metabolized in the liver into various metabolites upon absorption through oral ingestion, dermal absorption, and inhalation. DEHP is classified as a non-genotoxic hepatocarcinogen in rodents, as its chronic exposure has been associated with the development of liver cancer in these animals, but most genotoxicity studies have been negative. Epidemiologic studies in humans suggest that long-term high intakes of DEHP may be a risk factor for liver dysfunction.

The repeated-dose liver micronucleus (RDLMN) assay is a well-established method for assessing chromosomal changes caused by hepatic genotoxins and/or carcinogens. It is particularly valuable for detecting substances that undergo metabolic activation, especially when the metabolite has a short half-life or does not reach the bone marrow effectively. Therefore, we investigated whether the RDLMN assay could detect DEHP-induced micronucleus formation in the liver following a 14 or 28-day treatment.

**Results:**

We report that the RDLMN assay demonstrated an increased frequency of hepatic micronuclei in rats exposed to DEHP for 14 or 28 days. The increases in micronuclei correlated with hepatomegaly, an established response to phthalates in the liver. Conversely, no such increases were observed in the micronucleus assay using bone marrow from these rats.

**Conclusion:**

The detection of DEHP-induced micronuclei by the RDLMN assay suggests that this assay could detect the potential genotoxicity and hepatocarcinogenicity of DEHP. It also demonstrated the utility of the RDLMN assay in identifying metabolically activated hepatic carcinogens.

**Supplementary Information:**

The online version contains supplementary material available at 10.1186/s41021-024-00311-x.

## Introduction

Di(2-ethylhexyl) phthalate (DEHP; CAS No. 117–81-7) is a synthetic compound of the phthalate class that was first produced in the United States in 1939 [1]. Phthalates are widely used as plasticizers in the manufacture of polyvinyl chloride (PVC) products to enhance their flexibility and durability. These chemicals are non-covalently bound to plastics, leach out of products, and enter the body through oral ingestion, dermal contact, and inhalation. Upon absorption, DEHP is rapidly metabolized in the liver into various metabolites including the active metabolite, mono(2-ethylhexyl) phthalate (MEHP) [2, 3].

Despite numerous reports of its toxicity [4–6], DEHP remains one of the most commonly used phthalates in various consumer products and certain medical devices, such as intravenous bags, dialysis tubing, feeding tubes, and surgical gloves [7, 8]. Studies have indicated that DEHP is an environmental endocrine disruptor [4, 5, 9], and chronic exposure can have a range of effects on the human body, including liver toxicity, reproductive toxicity, neurotoxicity, and potential carcinogenicity [10–14].

Studies have provided evidence that chronic oral exposure of rodents to DEHP can lead to hepatomegaly and liver cancer, possibly by triggering peroxisome proliferation through activation of the peroxisome proliferator-activated receptor α (PPARα) [15]. More recently, a PPARα-independent pathways have also been proposed, suggesting that multiple pathways are involved in the effects of DEHP on the liver [16–18]. Genotoxicity tests have yielded mixed, but largely negative results (Table 1, [19–21]), suggesting that non-genotoxic mechanisms play a role in DEHP-induced hepatocarcinogenesis [22, 23]. Some of the positive results reported appear to be primarily related to oxidative DNA damage rather than direct DNA reactivity [24, 25]. The International Agency for Research on Cancer (IARC) has classified DEHP as a potential human carcinogen (Group 2B) [26]. In Europe, DEHP is listed as a Substance of Very High Concern (SVHC) primarily for its endocrine disrupting properties, while its carcinogenic potential has been noted [27, 28]. The carcinogenic potential of DEHP in humans remains an active area of research. In particular, epidemiologic studies are critical to understanding the long-term effects of DEHP exposure in human populations.

**Table 1:** Genotoxicity profiles and carcinogenicity data for DEHP

Ames test		in vitro mammalian cell tests		in vivo genotoxicity tests		Rodent carcinogenicity	
‒	[19]	‒	(MLA [19])	‒	(CA, CMT [19], UDS [20], TGR in liver [21])	+	[19]
		‒ / +	(CA [19])	‒ / + / E	(MN [19])		

*CA* chromosomal aberration test, *MLA* mouse lymphoma assay, *CMT* comet assay, *MN* peripheral blood/bone marrow micronucleus test, *UDS* unscheduled DNA synthesis test, *TGR* transgenic rodent gene mutation assay

‒: negative; + : positive; E: equivocal

The micronucleus (MN) assay stands as a sensitive and reliable method for evaluating chromosomal damage and estimating the carcinogenic potential of substances. Although bone marrow and peripheral blood are commonly used in the MN assay, the repeated-dose liver micronucleus (RDLMN) assay holds particular significance, especially for detecting substances with active metabolites [29]. The repeated-dose regimen used in the RDLMN assay overcomes the limitation of a low proliferation activity of hepatocytes (HEPs), rendering this assay highly sensitive for detecting both genotoxic and non-genotoxic hepatocarcinogens. It has been reported that genotoxic rodent hepatocarcinogens, which may require metabolic activation and may not be detectable by routine rodent erythrocyte micronucleus assays, can be identified using the liver micronucleus assay [29].

In this study, we evaluated the ability of the RDLMN assay to detect a MN induction by the hepatocarcinogen DEHP, which may require metabolic activation, while also using the erythrocyte MN assay for comparison.

## Materials and methods

### Animals

Male Crl:CD (SD) rats were purchased at 5 weeks of age from Charles River Japan Inc. (now The Jackson Laboratory Japan, Inc., Yokohama, Japan) and acclimated for 5 days. The animals were aged 6 weeks at the start of administration. The animals were housed individually in an air-conditioned room with a 12-h light/dark cycle and free access to food and drinking water. The experimental protocol was approved by the Institutional Animal Care and Use Committee prior to its implementation.

### Chemicals

DEHP (CAS No. 117–81-7, ≥ 98.0% purity) was purchased from Tokyo Chemical Industry Co., Ltd.

### Doses levels and treatment

The highest dose of DEHP was chosen in line with the highest dose in the 28-day repeated-dose study, 1000 mg/kg/day, as specified in the OECD guidelines (TG407). DEHP dosing formulations were prepared by suspending an appropriate amount of DEHP in the vehicle (corn oil, FUJIFILM Wako Pure Chemical Corporation, Japan) at the time of use. Animals were divided into four groups and received DEHP orally at 250, 500, and 1000 mg/kg/day, or vehicle alone (vehicle control group) at 10 mL/kg body weight once daily for 14 or 28 consecutive days.

### Liver micronucleus assay

The HEP suspension was prepared according to the method established by Narumi, et al., with slight modifications [30].

Twenty-four hours after the last administration for each time point, rats were euthanized under thiopental anesthesia.

Approximately 1 g of the left lateral lobe was sliced into several pieces that were 0.5–1 mm thick. The sliced tissues were rinsed with cold physiological saline (Otsuka Pharmaceutical Factory, Inc., Tokushima, Japan) and then treated with a digestion solution containing 100 units/mL of collagenase (Collagenase Yakult-S, Yakult Pharmaceutical Industry, Co., Ltd., Tokyo, Japan) to make a HEP suspension. Isolated HEPs were fixed with 10% neutral-buffered formalin, and the suspension was stored until microscopic observation. Immediately before observation, 10 μL of the HEP suspension was mixed with an equal volume of an acridine orange (AO: 0.5 mg/mL) and 4^′^,6-diamidino-2-phenylindole dihydrochloride (DAPI: 10 μg/mL) staining solution, dropped onto a glass slide, and covered with a coverslip.

All of the slide specimens were coded and scored blind during the specimen observation. The slide preparation was observed under a fluorescent microscope at 400 × magnification with U-excitation (ultraviolet ray excitation, wave length: 330 to 385 nm), and the number of micronucleated hepatocytes (MNHEPs) per 2000 parenchymal HEPs, including mono-, bi-, and multi-nucleate cells, was counted for each animal. MNHEPs were identified as HEPs with round or distinct micronuclei stained with the same color as the nuclei and with diameters of 1/4 or less than those of the main nuclei.

### Erythrocyte micronucleus assay

In contrast to the liver MN assay, the erythrocyte MN assay has been widely used as a routine method. Briefly, twenty-four hours after the last administration for each time point, rats were euthanized under thiopental anesthesia. After removing the livers, the femur was removed, and then erythrocytes were collected by washing the femur cavity with 10% neutral-buffered formalin. After centrifugation, the cells were fixed again with 10% neutral-buffered formalin and stored until analysis [31]. Immediately prior to the microscopic observation, the cells were mixed and stained with the same volume of AO (0.5 mg/mL). The stained cell suspension was dropped onto a glass slide and covered with a coverslip.

All of the slide specimens were coded and scored blind during the specimen observation. The specimens were observed under a fluorescent microscope at 600 × magnification with B-excitation (blue light excitation, wave length: 420 to 490 nm), and the number of micronucleated immature erythrocytes (MNIMEs) per 2000 immature erythrocytes (IMEs) was counted for each animal. As a parameter of the cytotoxicity, the ratio of IMEs to 1000 erythrocytes was also recorded for each animal.

### Liver weight measurement

On the day following the last dose, body weight and liver weight of each animal were measured at necropsy, and relative weights were calculated.

### Statistical analyses

Differences in the incidence of MNHEPs and MNIMEs between the test groups and the vehicle control group were analyzed statistically using Kastenbaum and Bowman’s tables. Since the variances were homogenous for the liver weight data and the ratio of IMEs to total erythrocytes by Bartlett’s test (5% level of significance), Dunnett’s test (5% level of significance) was performed to compare between each treated group and vehicle control group.

## Results

### DEHP induces micronuclei in the liver but not in bone marrow

**Fig. 1:**
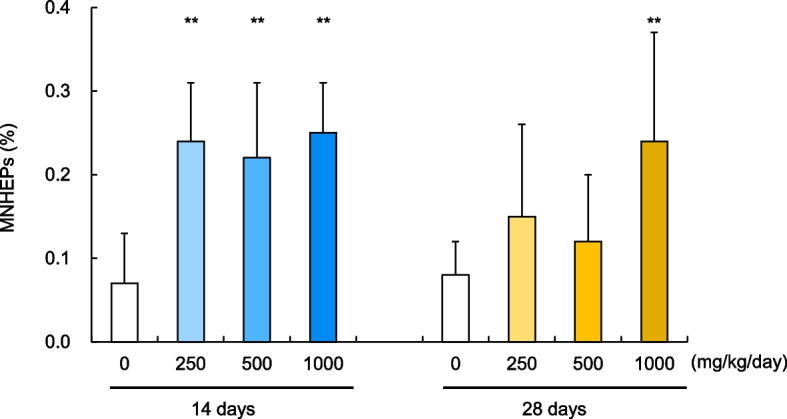
Comparative analysis of liver MN assay results in rats after 14 and 28 days of DEHP treatment. Incidence of MNHEPs (%); Comparison of results obtained after 14 and 28 days of repeated dosing with DEHP. Values are presented as the mean and SD. Differences in the incidence of MNHEPs between the test and vehicle control groups were analyzed at the 1% significance level (**: *P* < 0.01) using the Kastenbaum and Bowman test

**Fig. 2:**
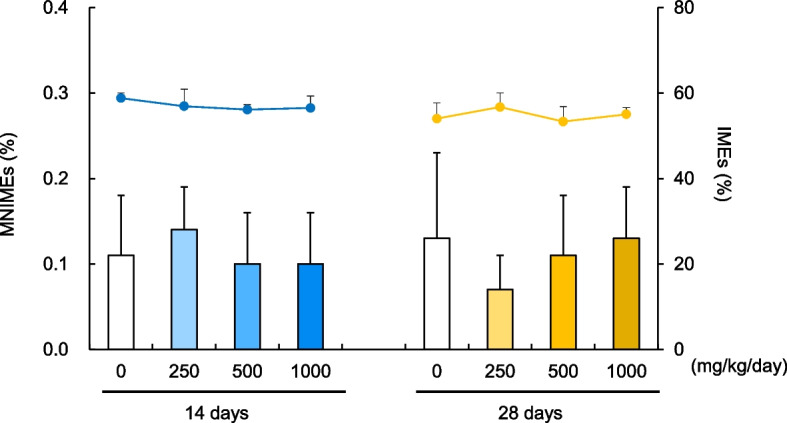
Comparative analysis of erythrocyte MN assay results in rats after 14 and 28 days of DEHP treatment. Incidence of MNIMEs (%) (bar graph) and ratio of IMEs to total erythrocyte (%) (line graph); Comparison of results obtained after 14 and 28 days of repeated dosing with DEHP. Values are presented as the mean and SD

We assessed MN frequencies in the HEPs of the liver and in immature erythrocytes in the bone marrow of the DEHP-treated animals and compared them to those in control animals. During the 14-day treatment regimen, we observed marked, statistically significant increases in MN frequencies in the HEPs of treated animals at all dose levels: 250, 500, and 1000 mg/kg/day (Fig. 1). The individual data and historical control data are given in additional files (see Additional files 1 and 2). In the 28-day treatment regimen, MN frequencies increased at all dose levels, with a statistically significant increase at the highest dose of 1000 mg/kg/day (Fig. 1). In contrast, no obvious micronuclei induction was observed in bone marrow regardless of the dose levels or the dosing periods (Fig. 2). The frequencies of immature erythrocytes in the bone marrow remained constant across all DEHP dose levels, indicating no treatment-associated cytotoxic effects on erythropoiesis.

### DEHP causes an increase in relative liver weight

**Fig. 3:**
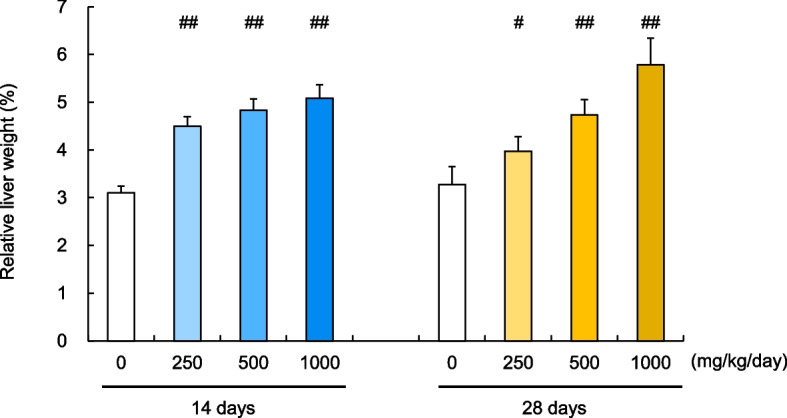
Comparative analysis of relative liver weights in rats after 14 and 28 days of DEHP treatment. Relative liver weights (%); Comparison of results obtained after 14 and 28 days of repeated dosing with DEHP. Values are presented as the mean and SD. Differences in the relative liver weights between the test and vehicle control groups were analyzed using Dunnett’s multiple comparison test at the 5% and 1% significance levels (#: *P* < 0.05, ##: *P* < 0.01).

Studies have shown that when a peroxisome proliferator is administered, peroxisome proliferation, hepatomegaly, and increased hepatocyte proliferation are observed in rodents via the activation mechanism of PPARα [15]. Following either a 14- or 28-day treatment with DEHP, a well-known peroxisome proliferator, the liver weights of the treated animals were, therefore, measured. As expected, the liver-to-body weight ratio significantly increased compared to the control animals in both treatment regimens, and these increases were dose-dependent (Fig. 3).

## Discussion

In the present study, we demonstrated that the RDLMN assay could efficiently detect the induction of micronuclei after repeated DEHP exposure to rats. This induction was not observed in bone marrow under the present conditions, suggesting that the RDLMN assay is highly sensitive to molecules that are metabolized and act in the liver, such as DEHP. Similar observations were made with clofibrate and methapyrilene HCl, non-genotoxic hepatocarcinogens, in the CSGMT/JEMS·MMS collaborative study [29]. The discrepancy between the RDLMN assay and the erythrocyte MN assay suggests that these metabolites may be short-lived or may not effectively reach the bone marrow. In the paper by Satomoto et al. [32], it was reported that the sensitivity of the liver micronucleus assay depends on the frequency of hepatocyte division, which decreases with age. However, in this study, the induction rate of hepatic micronuclei was lower in the 28-day repeated dose group than in the 14-day repeated dose group. This discrepancy could be explained by the fact that hepatic micronuclei are thought to be retained and accumulated for only about 14 days, and that the 14-day treated animals were 8 weeks old at the time of sacrifice, younger than the 10-week old animals treated for 28 days.

The fact that DEHP is known as a non-genotoxic rodent hepatocarcinogen raises an interesting question; the RDLMN assay may detect the compounds classified as non-genotoxic hepatocarcinogens. This is possible because micronucleus can be formed by several indirect mechanisms [33]. The exact mechanism of micronucleus induction by DEHP is not known but activation of PPARs, peroxisome proliferator, by DEHP is one of the possible mechanisms. PPARs are nuclear hormone receptors that regulate gene expression. One of the well-characterized effects of PPAR ligands, particularly those that bind to PPARα, is the proliferation of peroxisomes in cells [15]. An increase in the number of peroxisomes could lead to elevated levels of reactive oxygen species (ROS), which contributes to oxidative stress, leading in DNA damages [34]. If they are not adequately repaired, this can result in the formation of micronuclei. In fact, DEHP exposure has been associated with elevated levels of 8-hydroxydeoxyguanosine (8-OH-dG), a biomarker of oxidative stress and DNA damage [35]. Furthermore, ROS-induced lipid peroxidation can also damage DNA and other cellular components, potentially leading to micronucleus formation [36].

In addition, DEHP is a known endocrine disruptor that could interfere with hormone signaling pathways, which may indirectly lead to cellular stress or disruption of normal cell division, contributing to micronucleus formation [37, 38].

Furthermore, activation of PPARs could alter the expression of a wide range of genes, resulting in increased cell proliferation and decreased apoptosis [39]. This is consistent with the findings of hepatomegaly observed in this and other studies. Such changes can create a favorable environment for the progression of precancerous cells into malignant ones.

In the present study, we demonstrated that the RDLMN assay could efficiently and specifically identify carcinogenic potential of DEHP, an established rodent hepatocarcinogen.

## Conclusion

The detection of DEHP-induced micronuclei by the RDLMN assay suggests that this assay could detect the potential genotoxicity and hepatocarcinogenicity of DEHP. The high sensitivity of the RDLMN assay demonstrated the utility of the RDLMN assay in identifying metabolically activated hepatocarcinogens.

### Supplementary Information

Supplementary Material 1.

## Data Availability

All data generated or analyzed during this study are included in this published article.

## Abbreviations

8-OH-dG: 8-Hydroxydeoxyguanosine
AO: Acridine orange
CSGMT/JEMS·MMS: Collaborative Study Group of the Micronucleus Test/Japanese Environmental Mutagen Society Mutagenicity Study Group
DAPI: 4^′^,6-Diamidino-2-phenylindole dihydrochloride
DEHP: Di(2-ethylhexyl) phthalate
HEP: Hepatocyte
IME: Immature erythrocyte
MN: Micronucleus
MNHEP: Micronucleated hepatocyte
MNIME: Micronucleated immature erythrocyte
MEHP: Mono(2-ethylhexyl) phthalate
PPARα: Peroxisome proliferator-activated receptor α
PVC: Polyvinyl chloride
RDLMN: Repeated-dose liver micronucleus
ROS: Reactive oxygen species
SVHC: Substance of Very High Concern
