# Supplementary material for: Confirmation of Di(2-ethylhexyl) phthalate-induced micronuclei by repeated dose liver micronucleus assay: focus on evaluation of liver micronucleus assay in young rats
Source: Genes Environ. 2024 Aug 23;46:17. doi: 10.1186/s41021-024-00311-x (PMC11344444; doi:10.1186/s41021-024-00311-x)
Supplement: Supplementary file 1 — Supplementary Material 1. [file 41021_2024_311_MOESM1_ESM.pdf]

Additional file 1

Individual data of the liver and bone marrow micronucleus assay

Test chemical (mg/kg/day)		Number of animals	Liver	Bone marrow	
			MNHEPs (%)<br>Mean ± SD<br>(individual value)	MNIMEs (%)<br>Mean ± SD<br>(individual value)	IMEs (%)<br>Mean ± SD<br>(individual value)
DEHP (14 days)	0	5	0.07 ± 0.06<br>(0.05, 0.00, 0.10, 0.05, 0.15)	0.11 ± 0.07<br>(0.05, 0.15, 0.20, 0.10, 0.05)	58.8 ± 1.2<br>(59.1, 57.6, 60.8, 58.3, 58.4)
	250	5	0.24 ± 0.07<br>(0.20, 0.25, 0.35, 0.25, 0.15)	0.14 ± 0.05<br>(0.15, 0.05, 0.15, 0.20, 0.15)	56.9 ± 4.0<br>(56.9, 61.1, 55.7, 60.0, 50.9)
	500	5	0.22 ± 0.09<br>(0.25, 0.20, 0.10, 0.20, 0.35)	0.10 ± 0.06<br>(0.10, 0.00, 0.15, 0.15, 0.10)	56.1 ± 1.2<br>(57.2, 54.3, 56.9, 56.5, 55.6)
	1000	5	0.25 ± 0.06<br>(0.20, 0.25, 0.20, 0.35, 0.25)	0.10 ± 0.06<br>(0.10, 0.20, 0.10, 0.05, 0.05)	56.5 ± 2.8<br>(57.8, 56.7, 53.2, 60.3, 54.5)
DEHP (28 days)	0	5	0.08 ± 0.04<br>(0.05, 0.10, 0.15, 0.05, 0.05)	0.13 ± 0.10<br>(0.10, 0.10, 0.00, 0.25, 0.20)	54.0 ± 3.7<br>(52.6, 50.8, 51.6, 55.0, 60.1)
	250	5	0.15 ± 0.11<br>(0.30, 0.00, 0.15, 0.10, 0.20)	0.07 ± 0.04<br>(0.00, 0.10, 0.05, 0.10, 0.10)	56.7 ± 3.3<br>(55.3, 59.8, 60.7, 54.5, 53.3)
	500	6	0.12 ± 0.08<br>(0.05, 0.10, 0.05, 0.15, 0.25, 0.10)	0.11 ± 0.07<br>(0.20, 0.15, 0.05, 0.10, 0.15, 0.00)	53.3 ± 3.5<br>(51.2, 51.8, 55.8, 57.9, 54.7, 48.3)
	1000	5	0.24 ± 0.13<br>(0.35, 0.10, 0.10, 0.35, 0.30)	0.13 ± 0.06<br>(0.20, 0.15, 0.05, 0.10, 0.15)	55.0 ± 1.6<br>(53.9, 54.8, 56.1, 57.1, 53.2)

MNHEPs: micronucleated hepatocytes; MNIMEs: micronucleated immature erythrocytes.

Additional file 2

Historical control data of the liver and bone marrow micronucleus assay

Tissue	Duration	Number of animals	Frequency of MNHEPs/MNIMEs (%)	
			Mean ± SD (Mean ± 2SD)	Maximum and minimum individual value
Liver	14 days	48	0.07 ± 0.05 (0.07 ± 0.10)	Max: 0.20 Min: 0.00
	28 days	38	0.06 ± 0.05 (0.06 ± 0.10)	Max: 0.25 Min: 0.00
Bone marrow	14 days	25	0.14 ± 0.07 (0.14 ± 0.14)	Max: 0.30 Min: 0.00
	28 days	30	0.13 ± 0.08 (0.13 ± 0.16)	Max: 0.25 Min: 0.00

MNHEPs: micronucleated hepatocytes; MNIMEs: micronucleated immature erythrocytes.
